# Supplementary figures and images for: Nucleolin myocardial-specific knockout exacerbates glucose metabolism disorder in endotoxemia-induced myocardial injury
Source: PeerJ. 2024 May 20;12:e17414. doi: 10.7717/peerj.17414 (PMC11114111; doi:10.7717/peerj.17414)

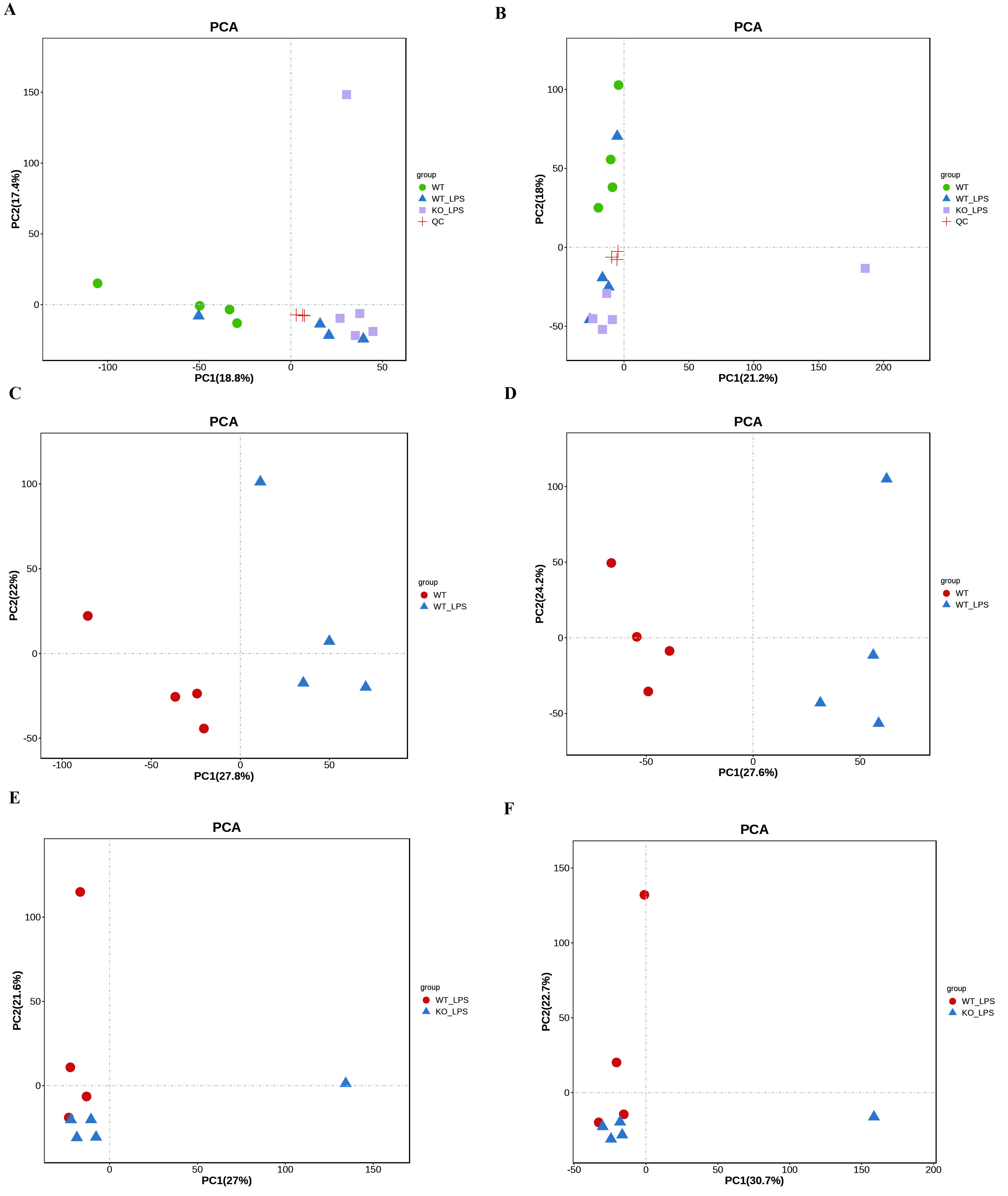

Supplement: Supplemental Information 1 [file peerj-12-17414-s001.zip › figure/FIGURE-1.jpg]

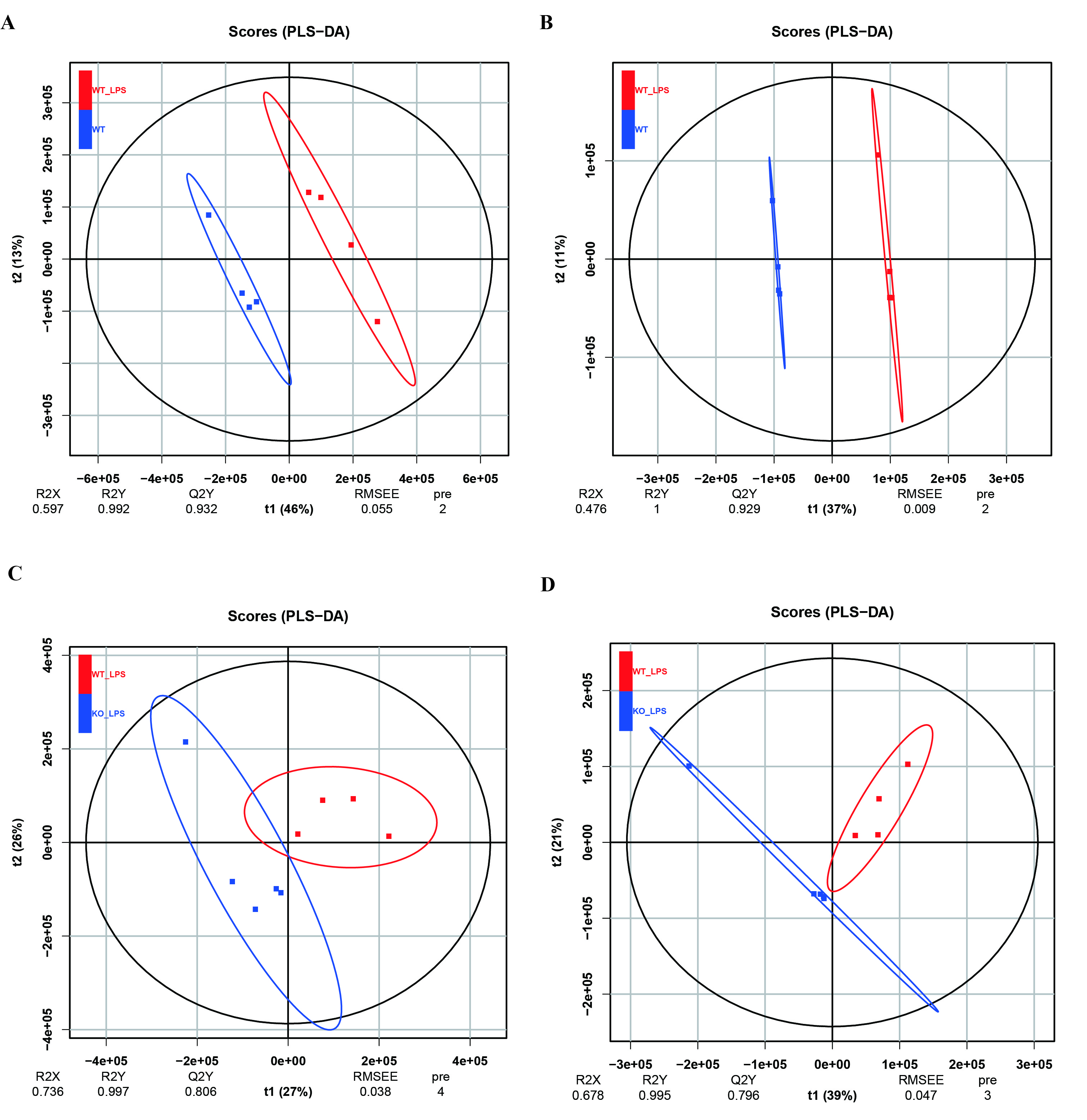

Supplement: Supplemental Information 1 [file peerj-12-17414-s001.zip › figure/figure-2.jpg]

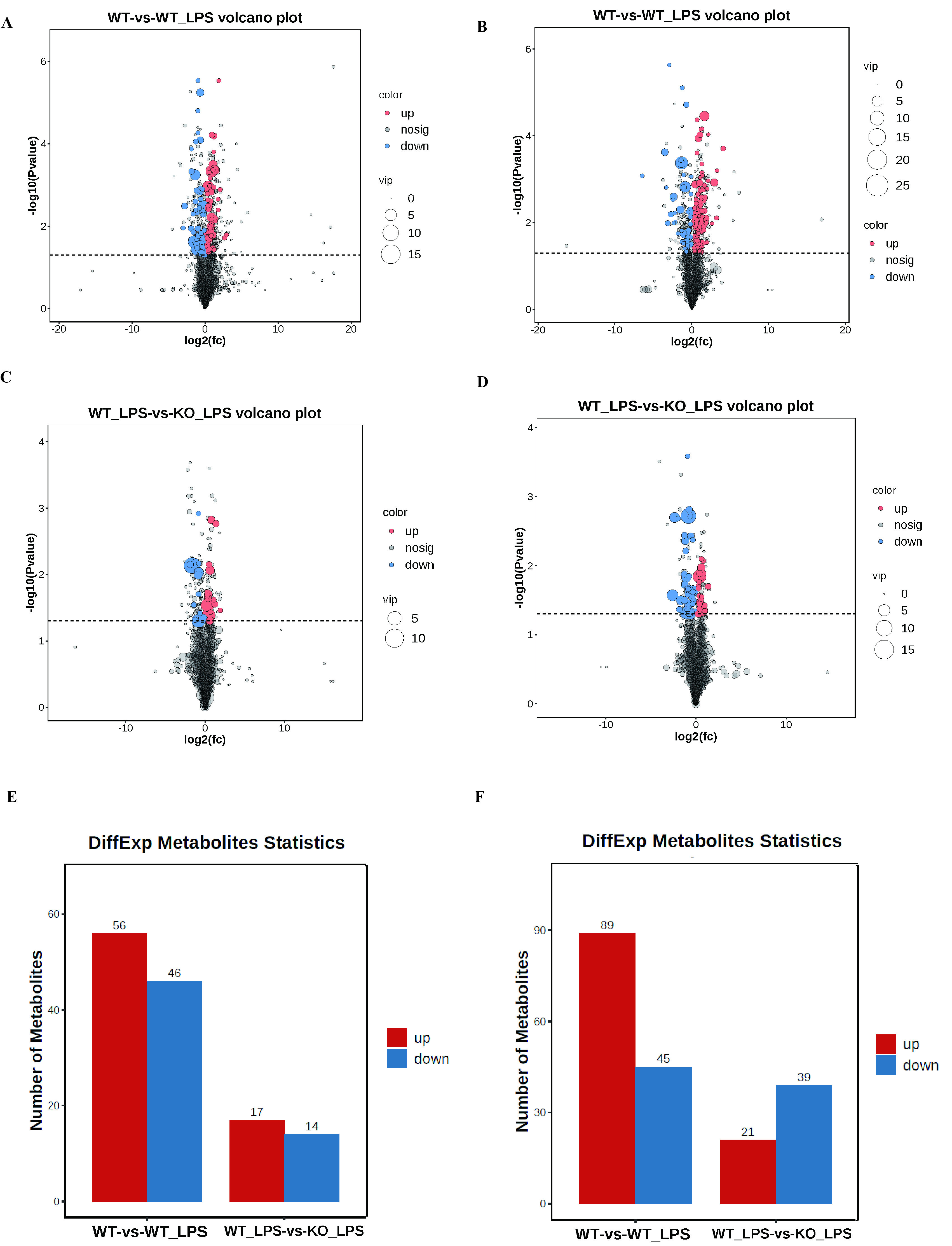

Supplement: Supplemental Information 1 [file peerj-12-17414-s001.zip › figure/figure-3.jpg]

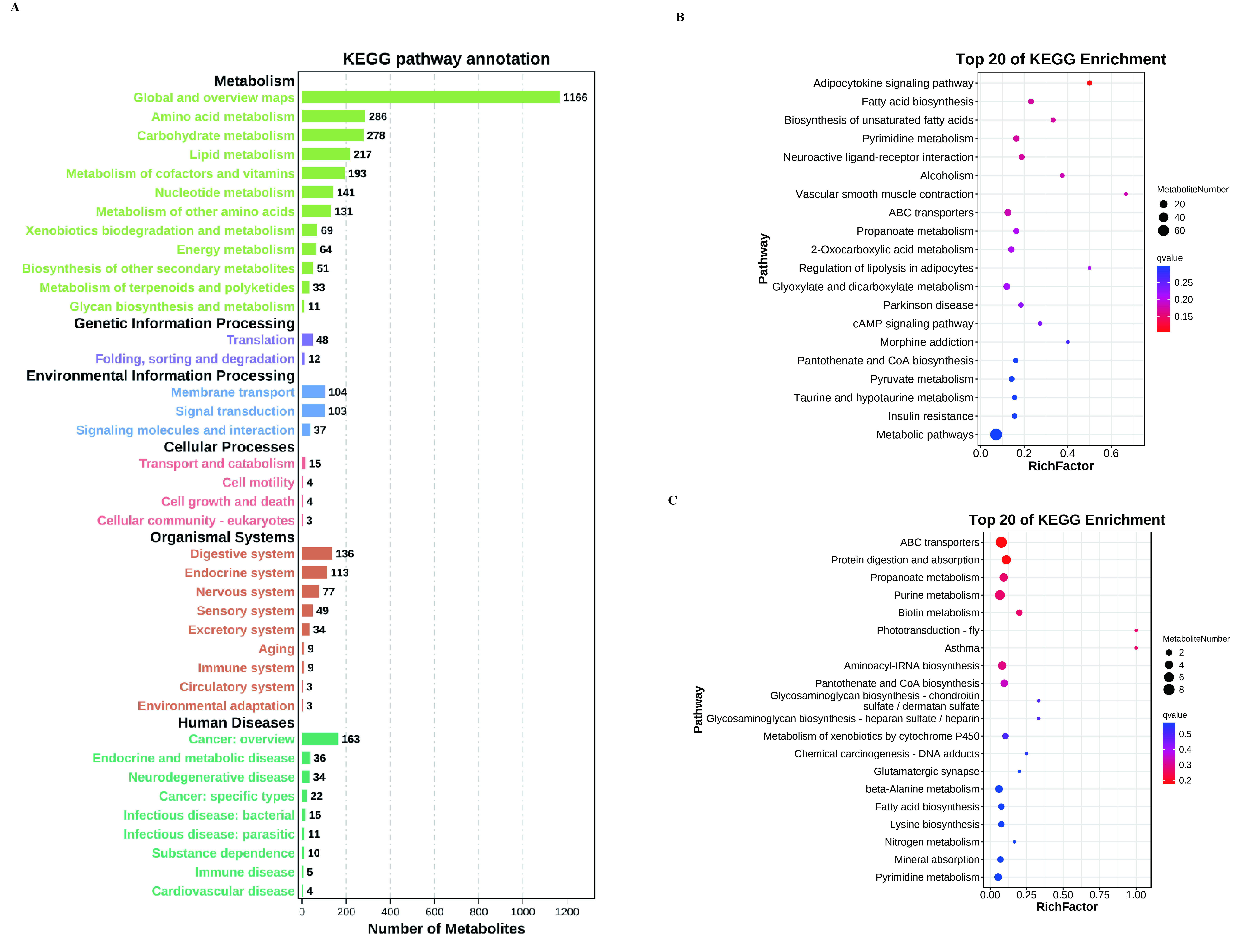

Supplement: Supplemental Information 1 [file peerj-12-17414-s001.zip › figure/figure-4.jpg]

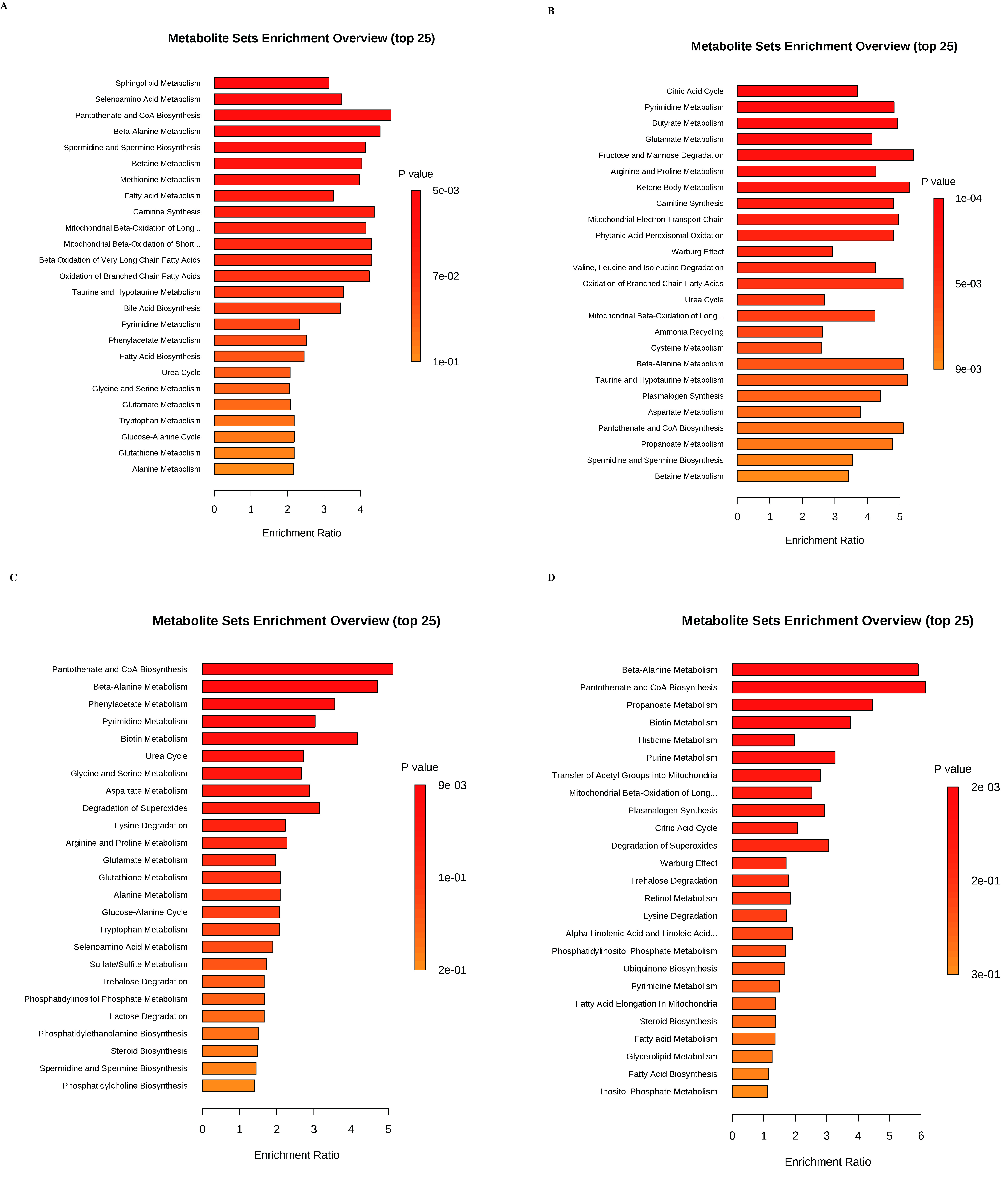

Supplement: Supplemental Information 1 [file peerj-12-17414-s001.zip › figure/figure-5.jpg]

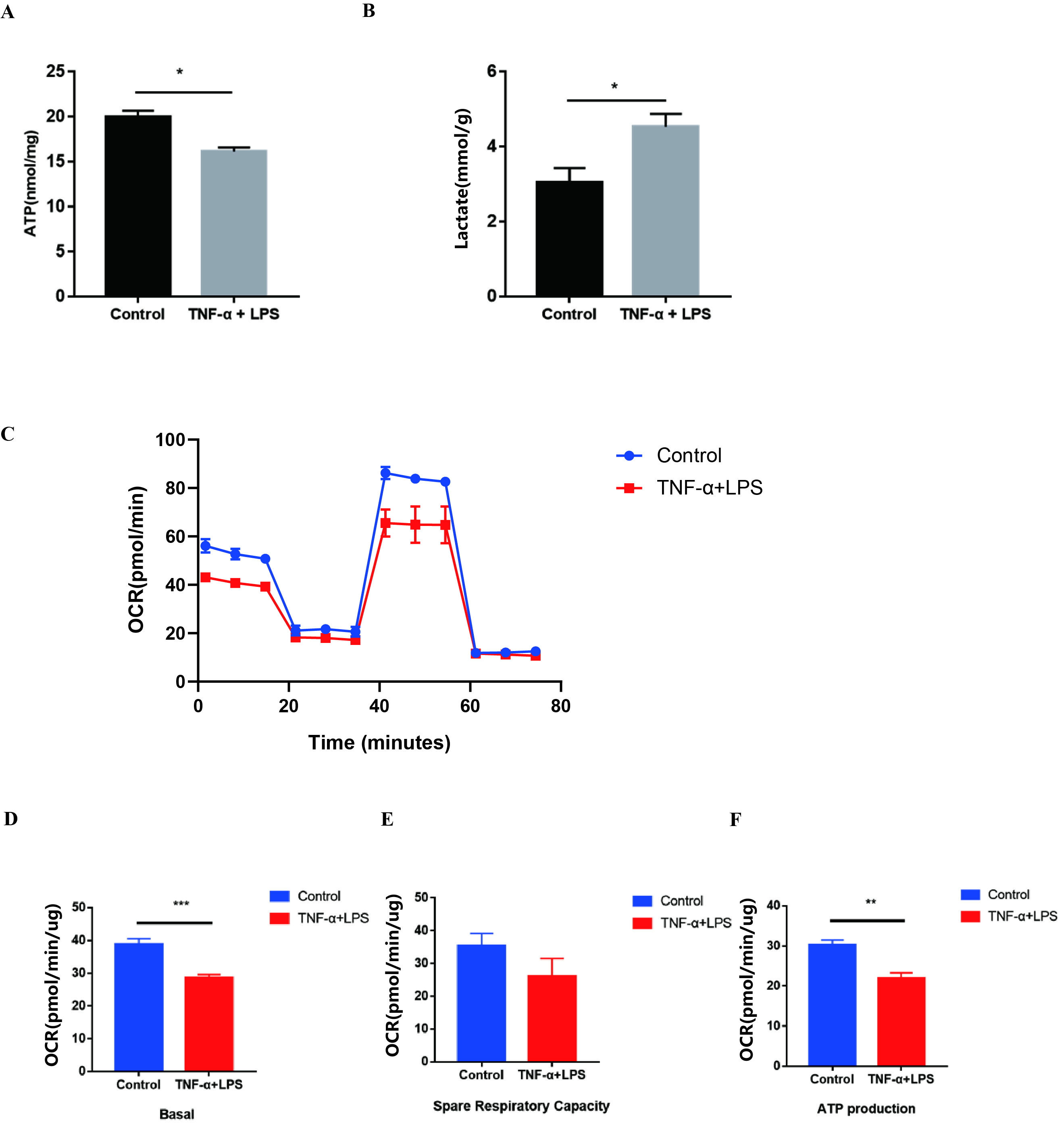

Supplement: Supplemental Information 1 [file peerj-12-17414-s001.zip › figure/figure-6.jpg]

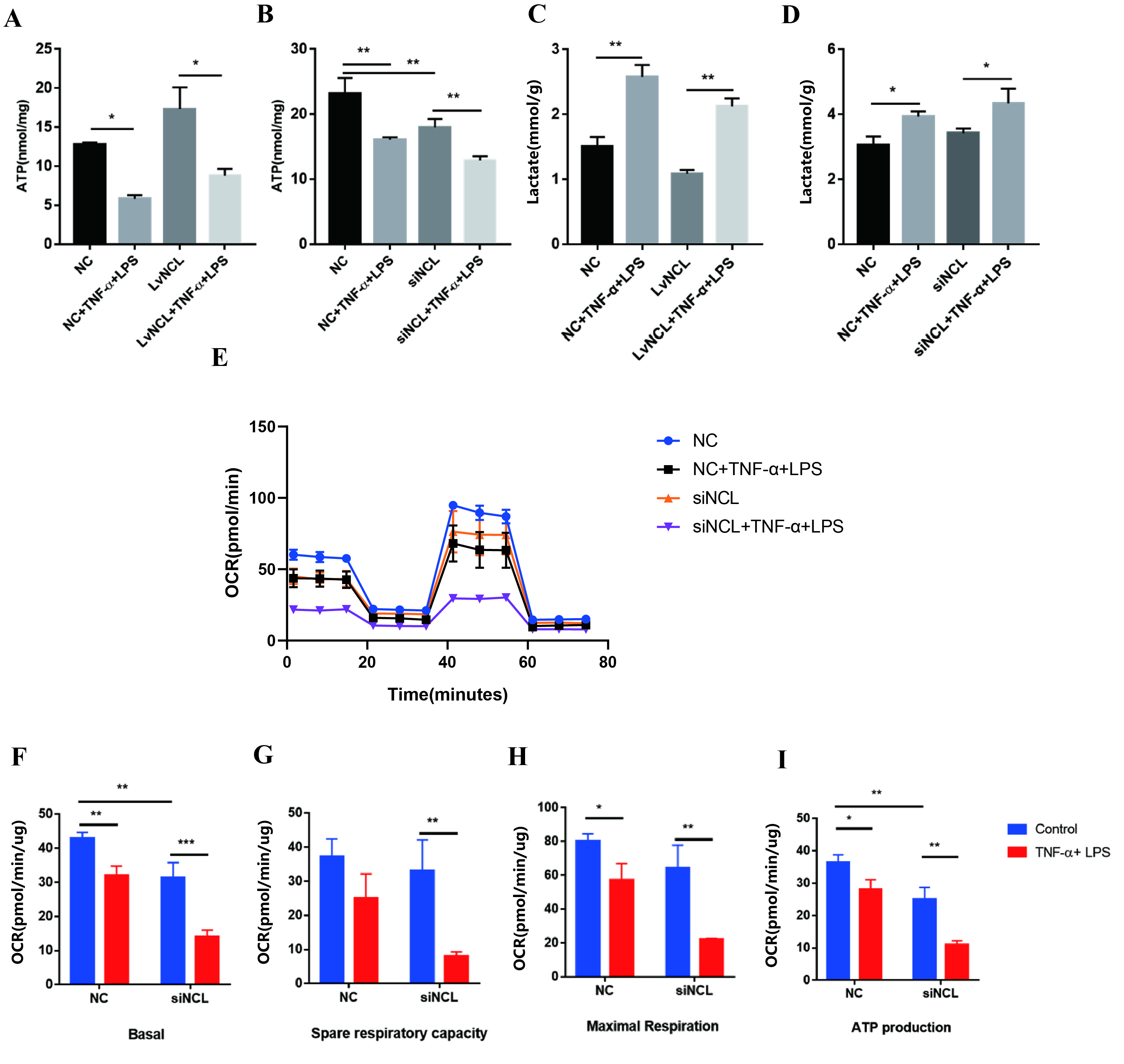

Supplement: Supplemental Information 1 [file peerj-12-17414-s001.zip › figure/figure-7.jpg]
